# Case Report: Retroaortic Innominate Vein With Supracardiac Total Anomalous Pulmonary Venous Connection

**DOI:** 10.3389/fped.2021.734567

**Published:** 2021-11-05

**Authors:** Toshinobu Ifuku, Ayako Kuraoka, Tomoko Ohhira, Koichi Sagawa, Toshihide Nakano, Hideaki Kado

**Affiliations:** ^1^Department of Pediatrics, Miyazaki Prefectural Miyazaki Hospital, Miyazaki, Japan; ^2^Department of Cardiology, Fukuoka Children's Hospital, Fukuoka, Japan; ^3^Department of Cardiovascular Surgery, Fukuoka Children's Hospital, Fukuoka, Japan

**Keywords:** congenital heart disease, cardiac surgery, total anomalous pulmonary venous connection, retroaortic innominate vein, echocardiogram, computed tomography

## Abstract

A retroaortic innominate vein (RAIV) is a rare anomaly that passes posterior to the ascending aorta to join the superior vena cava and is associated with congenital heart disease (CHD). The RAIV and normal left innominate vein (LIV) rarely duplicate. The etiology of the RAIV and its relationship with CHD remains unknown. We report a case involving a 1-month-old baby girl with RAIV and supracardiac total anomalous pulmonary venous connection (TAPVC). Transthoracic echocardiogram demonstrated a pulmonary venous confluence (CPV) posterior to the left atrium, an abnormal vertical vein (VV) that originated from the CPV, and a normally positioned LIV. Three-dimensional cardiac computed tomography revealed the VV and RAIV to which it merged. This is the first reported case of a combination of RAIV and isolated TAPVC. We speculate that the VV is connected to the CPV during fetal life, thus leaving the RAIV behind. The RAIV may be detected in various forms with the development of new diagnostic imaging methods. Although a RAIV itself does not require treatment, establishing a correct diagnosis before invasive tests and procedures are performed can help prevent unexpected complications.

## Introduction

Retroaortic innominate vein (RAIV) is a rare anomaly that passes posterior to the ascending aorta to join the superior vena cava (SVC) (1–3). RAIV occurrence is known to be associated with congenital heart disease (CHD) and, very rarely, with a normal heart structure (4, 5). The RAIV itself is asymptomatic and does not require treatment, but if its presence is not confirmed, problems may arise when invasive tests and treatments are performed.

Total anomalous pulmonary venous connection (TAPVC), on the other hand, is a typical cyanotic CHD in which all the pulmonary veins do not join the left atrium but instead join the systemic venous system. Pulmonary vein obstruction (PVO) may cause congestive heart failure and peripheral circulatory failure, requiring early cardiac surgery for TAPVC.

Herein, we report a case of RAIV associated with TAPVC in which pulmonary blood flowed through the RAIV. The combined occurrence of RAIV and isolated TAPVC has not yet been reported, and we believe that structure and hemodynamics observed are suggestive from an embryological perspective.

## Case Report

A 1-month-old girl with central cyanosis was referred to our institution for further investigation. The perinatal period was unremarkable, with vaginal delivery at term. At birth, the girl weighed 3.0 kg and had an Apgar score of 8. She was discharged from the obstetrician's office after birth; however, cyanosis remained undetected. No family history of genetic abnormalities were noted.

On admission, a physical examination revealed no symptoms of respiratory distress or heart murmur. Oxygen saturation was 85–90% in room air, blood pressure in the right upper extremity was 109/72 mmHg, heart rate was 143 beats per minute, respiratory rate was 48 breaths per minute, and body temperature was 36.2°C.

**Figure 1:**
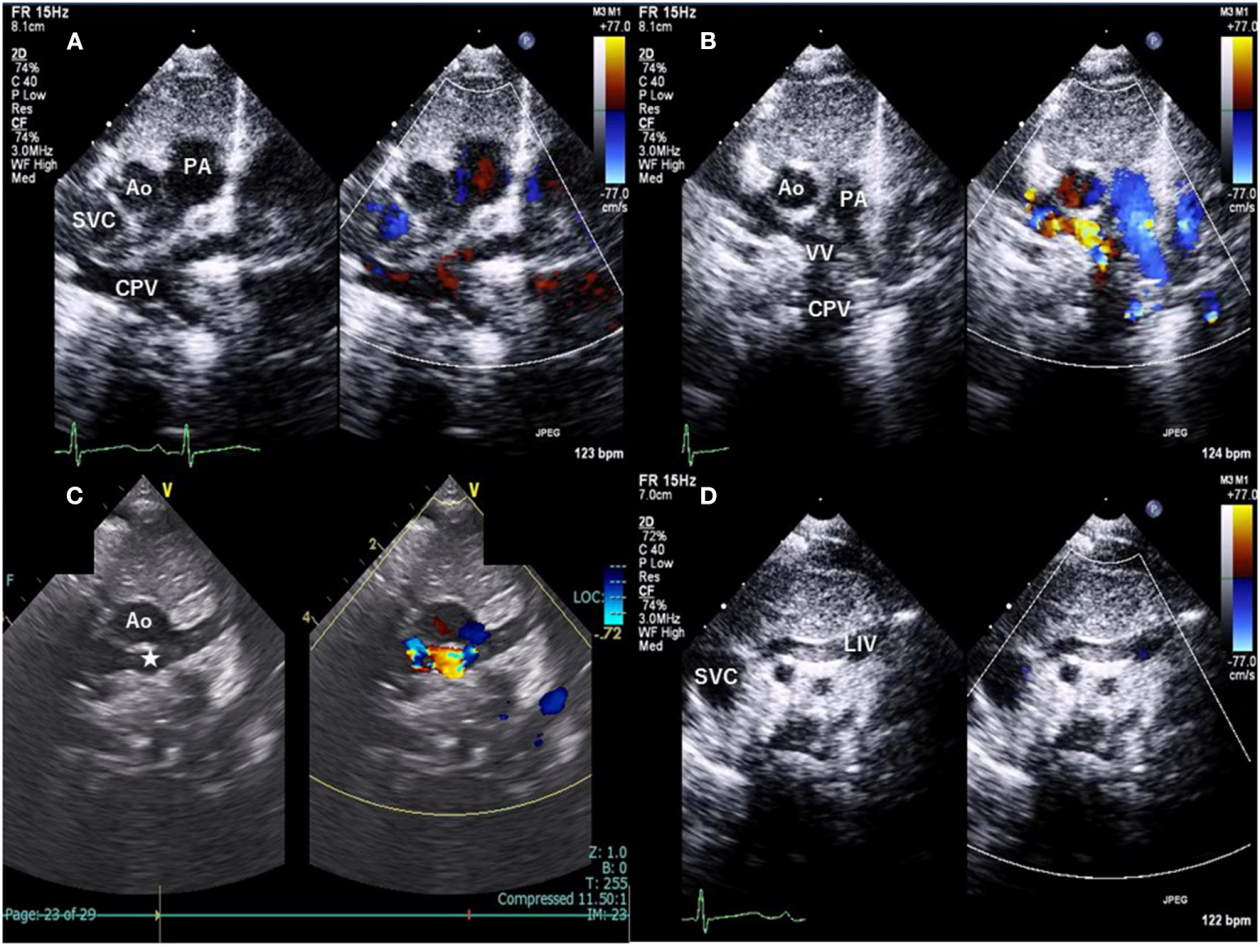
Suprasternal notch view of transthoracic echocardiography with color Doppler. **(A)** The pulmonary venous confluence (CPV) posterior to the left atrium. **(B)** The vertical vein (VV) originating from the CPV. **(C)** The anomalous vessel (asterisk) flowing from left to right behind the ascending aorta. **(D)** The left innominate vein in its normal position is not so dilated. Ao indicates aorta; PA, pulmonary artery; SVC, superior vena cava; LIV, left innominate vein.

A transthoracic echocardiogram demonstrated a right-sided chamber enlargement and a right-to-left shunt across the interatrial septum. The suprasternal notch view showed pulmonary venous confluence (CPV) posterior to the left atrium (LA) and a vertical vein (VV) that originated from the CPV (Figures 1A,B). Additionally, an anomalous vessel flowing from left to right behind the aorta was observed, and the left innominate vein (LIV), which was positioned normally, was not so dilated (Figures 1C,D). However, this test alone was not enough to fully understand the structure and positioning of the blood vessels.

**Figure 2:**
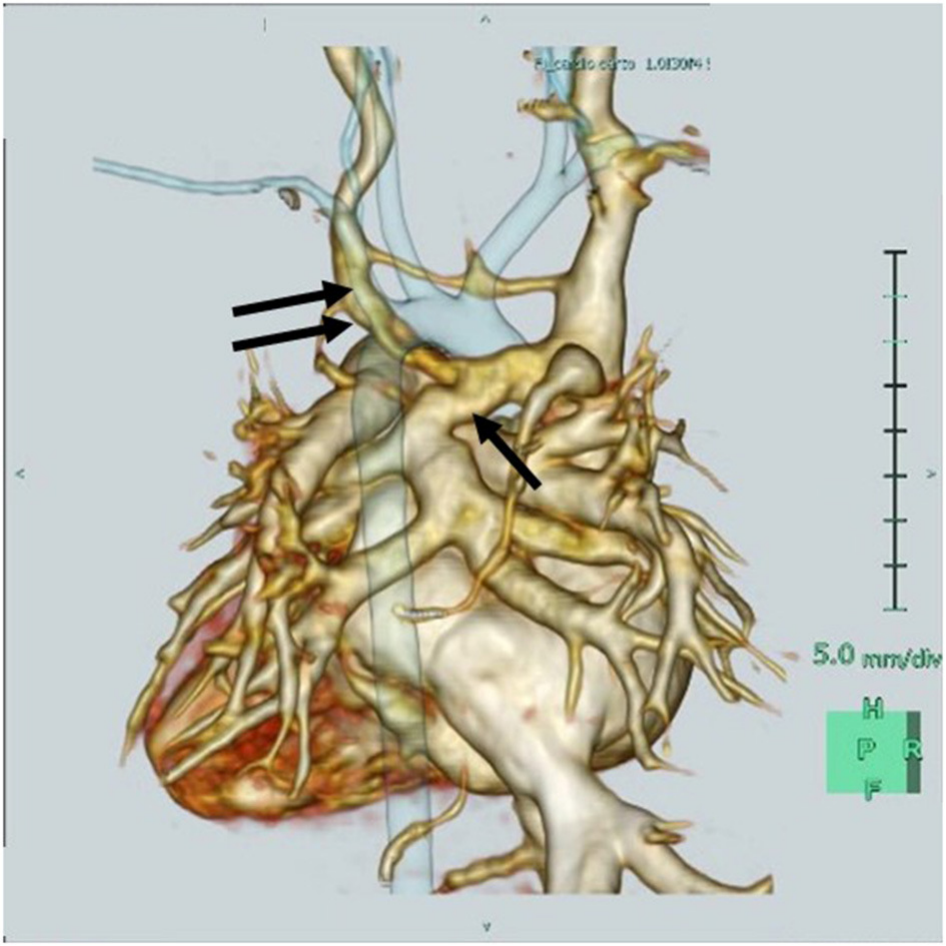
Three-dimensional cardiac computed tomography reveals that the retroaortic innominate vein (RAIV, double arrow) passes posterior to the ascending aorta to join the superior vena cava and the vertical vein (single arrow) merges into the RAIV.

A three-dimensional cardiac computed tomography confirmed the RAIV had dilated, and the VV that merged with the RAIV (Figure 2). The patient was finally diagnosed with supracardiac TAPVC complicated with RAIV.

Surgical repair was immediately performed with median sternotomy. Cannulation of the SVC was performed at the distal part of the LIV using a 10Fr angled catheter. After cardiopulmonary bypass and cardioplesic arrest, the VV was ligated by approaching from the right side and incision was made in the CPV. The CPV was anastomosed to the LA with running 6-0 Prolene sutures (Ethicon, Somerville, NJ). Finally, the atrial septal defect was patch-closed. The duration of aortic cross-clamp and perfusion time was 167 and 79 min, respectively.

The surgery was successful, and allowed hemodynamics to normalize, and cyanosis to disappear. The patient developed atrial tachycardia on postoperative day 6, and the arrhythmia gradually corrected, with intravenous administration of landiolol (1–7 mcg/kg per minute) and procainamide (5–10 mg/kg). No postoperative infection occurred.

She was discharged 1 month postoperatively, with no restrictions in the activities of daily living and no significant problems with her postoperative course. She has been followed up with regular physical examinations, chest radiography, electrocardiography, and echocardiography, and no significant sequelae, such as PVO or severe supraventricular arrhythmias, have been noted.

The patient's parents provided informed consent for publication of this report.

## Discussion

This case has two key clinical implications. First, the RAIV can be further complicated by different CHDs and diverse morphologies. Second, prior recognition of the RAIV in patients who require invasive treatment is important.

The exact etiology of the RAIV remains unknown. Normally, the LIV is formed during fetal life from an inter-anterior cardinal anastomosis connecting the left and right anterior cardinal veins. Of the transverse anastomoses, the upper capillary plexus develops the future LIV, and the lower capillary plexus regresses. RAIVs are formed owing to failure of usual development of the upper transverse anastomosis either a partially leftover lower transverse anastomotic channel or an overexpression of the channel (2, 3, 6–8). The development of the upper capillary plexus can be interfered if there is an abnormality position of the vessels, such as the right-sided aortic arch (1, 8). Some researchers believe that if the aortic arch is in a high position or if there is adequate space under the aortic arch, a RAIV may form (9). As in this case, a RAIV and normal LIV very rarely duplicate.

Conversely, in supracardiac TAPVC, the VV from the CPV typically enters either via the LIV or SVC, or occasionally, the azygos vein (10). This case is believed to be a result of the VV connecting to a nearby RAIV. The pulmonary blood flowed into the RAIV; therefore, instead of the RAIV dilating, the normal LIV was not so much.

TAPVC requires surgical repair as soon as it is diagnosed. Although the cardiovascular structure and hemodynamics normalize after surgery, PVO due to stenosis at the LA and CPV anastomoses or supraventricular tachycardia may develop postoperatively. Since 1998, the sutureless technique has been used as the initial repair method for TAPVC (11). In this technique, the CPV is not anastomosed directly to the LA, but the posterior pericardium is anastomosed to the LA to form a new LA. The sutureless technique has been reported to have a lower risk of postoperative PVO and reoperation compared to conventional repair (12). In this case, the body weight and size of the CPV were sufficient, so the surgeon chose conventional repair, which he had enough experience with. However, for patients with low body weight, hypoplastic CPV, mixed type TAPVC, and right isomerism complications, we choose the sutureless technique.

A RAIV is present in 0.2–1% of patients with CHD, and most are associated with tetralogy of Fallot and a right-sided aortic arch (1–4). To the best of our knowledge, only one case of RAIV complicated with TAPVC has been reported in the past (13). Leal et al. found that among 9,897 patients, 14 had a RAIV. One of the patients had right isomerism and TAPVC with complex cardiac malformations, i.e., a hypoplastic left ventricle, a double inlet right ventricle through a common atrioventricular valve, and large atrial and ventricular septal defects. The VV flowed into RAIV, as was observed in our case, and the exact diagnosis of RAIV was made by echocardiography, surgery, and necropsy. This is the first case of a RAIV associated with isolated TAPVC. The RAIV and LIV duplicated, making it difficult to accurately identify the site and vascular structures associated with VV inflow.

**Figure 3:**
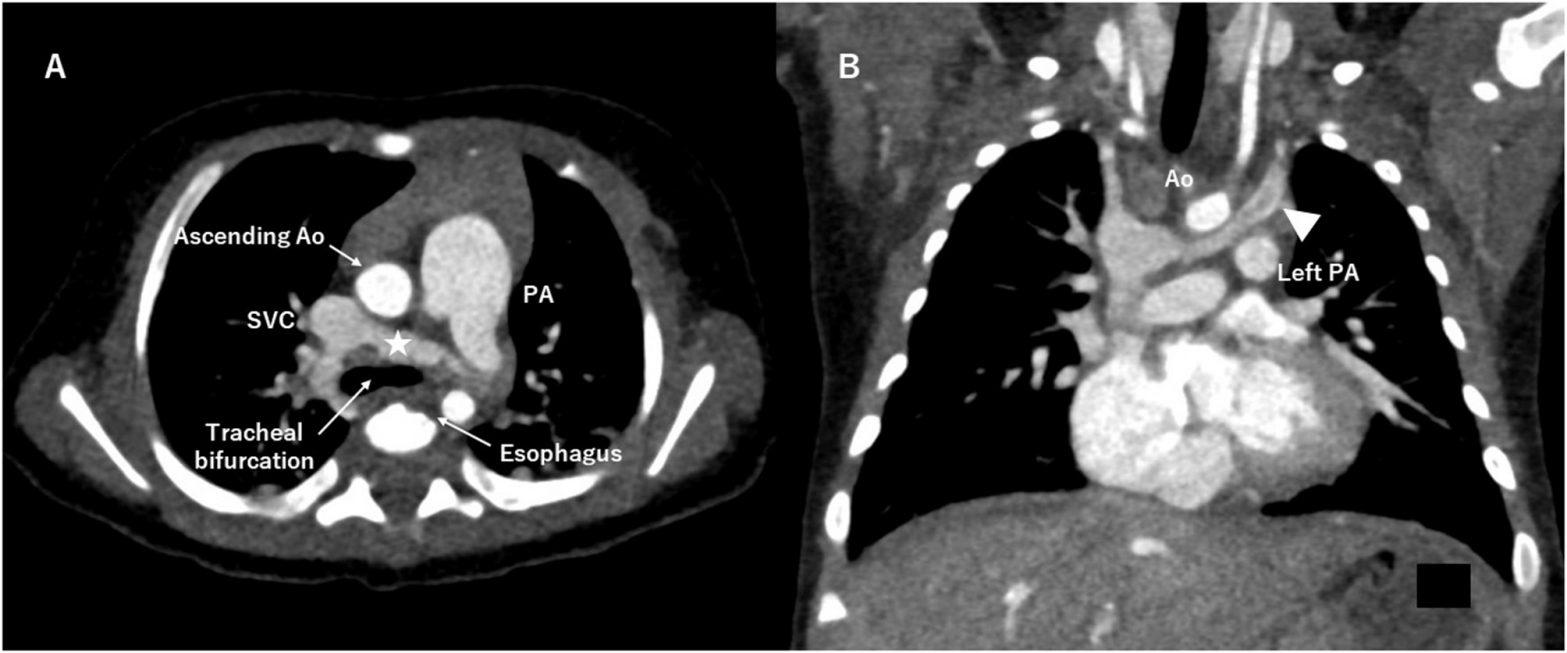
Computed tomography angiography. **(A)** The retroaortic innominate vein (RAIV, asterisk) runs near the tracheal bifurcation and the esophagus. **(B)** The descending portion of the RAIV (arrowhead) is similar to a persistent left superior vena cava. Ao indicates aorta; PA, pulmonary artery; SVC, superior vena cava.

A RAIV itself does not require treatment, but its identification is important, particularly in patients who require cardiac surgery, cardiac catheterization, pacemaker implantation, or central venous line placement. Because the RAIV is located posterior to the ascending aorta and near the trachea and esophagus, its presence may limit the surgical view and confuse the anatomy (Figure 3A). Lim et al. pointed out that the descending portion of the RAIV may be confused with a persistent left SVC (Figure 3B) and may interfere with securing an appropriate surgical field and cannulation for systemic venous drainage (4). In our case, accidental surgical ligation of the RAIV could have proved disastrous. The differential diagnosis of the RAIV includes a persistent left SVC, left partial anomalous pulmonary venous connection, patent ductus arteriosus, and aorto-pulmonary collateral artery. The echocardiographic and color Doppler findings as well as three-dimensional images confirmed the location of the abnormal vessels and the direction of blood flow and excluded these differential diagnoses.

In summary, we report the first case of a RAIV associated with isolated supracardiac TAPVC. A RAIV is a rare anomaly, but correct diagnosis before invasive tests and procedures can help prevent unexpected complications. The formation of CPV during fetal life may have influenced the development or persistence of RAIV. By presenting such a rare disease, we hope that the association between RAIV and CHD will be further investigated in terms of embryology and hemodynamics.

## Data Availability Statement

The original contributions presented in the study are included in the article/supplementary material, further inquiries can be directed to the corresponding author/s.

## Ethics Statement

Ethical review and approval was not required for the study on human participants in accordance with the local legislation and institutional requirements. Written informed consent was obtained from the individual(s), and minor(s)' legal guardian/next of kin, for the publication of any potentially identifiable images or data included in this article.

## Author Contributions

TI: cared for the patient, conceptualized the report, reviewed the cases, and drafted the manuscript. AK, TO, and HK: cared for the patient, reviewed the cases, collected data, and drafted the manuscript. TN and KS: collected data and drafted and revised the manuscript. All authors approved the final manuscript as submitted.

## Conflict of Interest

The authors declare that the research was conducted in the absence of any commercial or financial relationships that could be construed as a potential conflict of interest.

## Publisher's Note

All claims expressed in this article are solely those of the authors and do not necessarily represent those of their affiliated organizations, or those of the publisher, the editors and the reviewers. Any product that may be evaluated in this article, or claim that may be made by its manufacturer, is not guaranteed or endorsed by the publisher.
